# Comparative Study of Hypo-Fractionated Radiotherapy Versus Conventional Radiotherapy in Breast Cancer

**DOI:** 10.7759/cureus.29147

**Published:** 2022-09-14

**Authors:** Abhinav Narwariya, Mhendr Dhakar, Jagannath Jatav, Raju Prajapati, Sujata Bayer, Shajiya sarwar Moosa, Mohammad habeebur raheman Shaikh, Mohammed Taher Ali, Arafat Ahmad, Yousuf Begum, Sanket D Hiware

**Affiliations:** 1 Medical Oncology, Apollo Hospitals, New Delhi, IND; 2 Radiation Oncology, All India Institute of Medical Sciences, Raipur, Raipur, IND; 3 Pathology, Shyam Shah Medical College and Sanjay Gandhi Memorial Hospital, Rewa, IND; 4 Radiation Oncology, Shyam Shah Medical College and Sanjay Gandhi Memorial Hospital, Rewa, IND; 5 Anatomy, Imam Abdulrahman Bin Faisal University, Dammam, SAU; 6 Clinical Pharmacology, Imam Abdulrahman Bin Faisal University, Dammam, SAU; 7 Biochemistry, Imam Abdulrahman Bin Faisal University, Dammam, SAU

**Keywords:** breast cancer radiotherapy, hypofractionation, lumpectomy, mastectomy, conventional radiotherapy

## Abstract

Background: Breast cancer is the most commonly diagnosed cancer causing death among females worldwide. Radiotherapy after lumpectomy/mastectomy in breast cancer cases is a successful treatment modality taking five weeks to complete. The aim of the present study is to compare the effectiveness of hypo-fractionated radiotherapy in breast cancer patients with conventional radiotherapy with respect to outcome and toxicity.

Methods: Sixty patients were randomly divided equally into a conventional group, Group A (dose: 50 Gy in 25 fractions), and a hypo-fractionated short-course radiotherapy group, Group B (dose: 40 Gy in 16 fractions). After thorough clinical and laboratory examination of all patients, the disease status was assessed prior to radiotherapy and three and six months after completion of radiotherapy. The cardiopulmonary function was assessed using echocardiography and pulmonary function tests prior to the procedure. The assessment of the development of toxicity (dysphagia, skin, lung, and lymphedema) was done during every clinical visit.

Results: The mean age of patients was 53.28 ± 9.73 years in Group A, and 55.67 ± 10.41 years in Group B (p=0.82). The right breast was involved in 13 (43.4%) patients in Group A and 14 (46.6%) in Group B, and the left breast was involved n 17 (56.6%) patients in Group A and 16 (53.4%) in Group B (p=0.81). Most of the patients were post-menopausal; 24 (80%) in Group A and 25 (83.4%) in Group B (p=0.91). Eleven (36.6%) patients were of stage T2N1M0 in both groups. However, no statistical difference was observed between the groups in the TNM (tumor, node, and metastasis) staging using the AJCC (American Joint Committee on Cancer) criteria (p=0.26). On comparing the responses in Group A and Group B, no significant difference was observed in either of the groups from immediate post-treatment to the 12-month follow-up period (p=0.53 and p=0.64, respectively).

Conclusion: Hypo-fractionated radiotherapy is as effective as conventional radiotherapy and can be used as an alternative method for treatment following breast cancer surgery.

## Introduction

The terms “neoplasia” and “neoplasm” are difficult to define accurately. Mallon et al. suggested that “a neoplasia is a new growth, comprising an abnormal collection of cells, the growth of which exceeds and is uncoordinated with that of the normal tissue" [1]. Breast cancer is the most frequently diagnosed and the leading cause of global cancer incidence, accounting for 11.7% of all cancer cases. In India, breast cancer accounts for 13.5% of all cancer cases, accounting for 178,361 new cases and 90,408 deaths in 2020 [2]. The important risk factors for breast cancer are age over 50 years, early menarche and late menopause, family history (for women with a history in a first-degree relative, the risk is 1.7 to 2.5), older age at first childbirth, exogenous hormone, benign breast disease, radiation exposure, high body mass index, high fat diet, alcohol consumption, and high breast density [3]. Screening test for breast cancer includes mammography, magnetic resonance imaging, ultrasound, and physical examination [4,5].

Bone is the most common site of metastatic recurrence in breast cancer and the first site of metastases in more than 50% of the patients who fail systemically [6]. Breast cancer is mainly sub-classified by estrogen receptor (ER), progesterone receptor (PR), and human epidermal growth factor receptor 2 (HER2) neu status and these are strongly related to the pattern of metastases, in terms of site-specific relapse, early/late metastasis, survival outcomes, and prognosis. Hormone receptor-positive (ER, PR) tumors are more likely to spread to bone as a primary site of metastases and they have better survival outcomes as compared to the HER2 neu-positive tumors, which have a tendency of developing visceral metastasis [7].

The present study compares the tolerance of hypo-fractionated radiotherapy after breast conserva­tive surgery (BCS) or post-mastectomy using a regimen of 40 Gy (Gray) in 16 fractions in three weeks with conventional radiotherapy in a control group of 50 Gy in 25 fractions over five weeks. The primary objective of the study was to assess acute and chronic toxicity in hypo-fractionated versus conventional radiotherapy arm while the secondary objective was to compare the outcome of hypo-fractionated and conventional radiotherapy in breast cancer.

## Materials and methods

This randomized controlled trial was conducted in the Department of Radiotherapy, Gandhi Medical College and associated Hamidia Hospital, Bhopal, Madhya Pradesh, India, and Jawaharlal Nehru Cancer Hospital, Bhopal, Madhya Pradesh, India, over a period of 20 months from October 2016 to June 2018. The patients were randomly divided into a conventional treatment group, Group A (dose: 50 Gy in 25 fractions), and a hypo-fractionated short-course radiotherapy group, Group B (dose: 40 Gy in 15 fractions). Sixty patients were randomized into two equal groups using the chit-box method. Written informed consent was obtained from all patients and the Institutional Ethics Committee of Gandhi Medical College approved the study protocol (letter no: GMC/IEC/AS24).

Inclusion criteria comprised all histologically proven postoperative breast cancer (infiltrating ductal carcinoma) cases who underwent BCS without any evidence of distant metastasis, aged 18-75 years, Karnofsky Performance Scale (KPS) score of more than 70, and normal cardiac and pulmonary functions. Exclusion criteria comprised advanced stages of breast cancer where radiotherapy was not indicated, palliative radiotherapy, KPS score less than 70, uncontrolled hypertension and diabetes mellitus, distant metastasis, and co-existing heart disease and chronic pulmonary disorders.

Response evaluation was done after four to six weeks of completion of chemoradiotherapy and thereafter followed up every one to three months. Suspicious residual or recurrent lesions were confirmed by needle or tissue biopsy. Evaluation was according to Response Evaluation Criteria In Solid Tumors (RECIST) criteria as having a complete response, partial response, stable disease, and progressive disease. Patients with residual or recurrent disease were offered salvage chemotherapy or possible surgical intervention or palliative treatment.

After thorough clinical and laboratory examination of all patients, the disease status was assessed prior to radiotherapy and three and six months after completion of radiotherapy. Heart function was assessed by measuring the left ventricular ejection fraction (LVEF) by echocardiography. Pulmonary function tests were assessed by forced expiratory volume in one second (FEV1), forced vital capacity (FVC1), and the ratio of FEV1/FVC1. The assessment of the development of toxicity (dysphagia, skin, lung, and lymphedema) was done during every clinical visit. The scoring of toxicities was based on the common terminology criteria of adverse events by the Radiation Therapy Oncology Group (RTOG) and the worst grade was reported.

The biplane method of disks (modified Simpson method) is a two-dimensional (2D) echocardiographic technique requiring area tracings of the left ventricular (LV) cavity. This is the method recommended by the American Society of Echocardiography for measuring LVEF. It requires tracing the LV endocardial border in the apical four-chamber and two-chamber views in both end-diastole and end-systole. The tracings are used to divide the LV cavity into a predetermined number of disks (usually 20) with disk volumes based on the tracings. 

Dosimetric analysis

Patients were treated either with single‑beam energy or a combination of both 6 and 15 MV beams depending on their anatomy. The treatment was planned with a goal of 100% volume of planning target volume (PTV) to be covered by 95% isodose line. Data collected included the volume of PTV receiving at least 95% and 90% of prescribed dose (V95 and V90) and also dose delivered to 90% of the volume of PTV (D90%) from the dose‑volume histograms. The acceptable hot spot limit was 107%. The treatment plan was accepted if the volume of heart receiving 25Gy was ≤10% (i.e. V25 heart ≤10%) and the volume of ipsilateral lung receiving 20 Gy was ≤35% (i.e. V20 ipsilateral lung ≤35%).

Statistical analysis

The recording of data was done on Microsoft Excel 2007 (Microsoft Corporation, Redmond, Washington, United States) and the analysis was performed using IBM SPSS Statistics for Windows, Version 21.0 (Released 2012; IBM Corp., Armonk, New York, United States). All non-parametric data were analyzed using the Chi-square test. Student’s t-test or ANOVA was used for the analysis of parametric data. P-value less than 0.05 was considered significant.

## Results

All patients were successfully recruited into the study with no dropouts. Sixty patients were randomized into two groups; the mean age of patients was 53.28 ± 9.73 years in Group A, and 55.67 ± 10.41 years in Group B (p=0.82) (Table 1). On comparing BMI between the groups, no significant difference was observed (p=0.79). Neoadjuvant chemotherapy (NACT), adjuvant chemotherapy, and hormonal therapy were given to 56.6%, 90%, and 60% of patients, respectively, in Group A and 50%, 93.3%, and 63.3%, respectively, of patients in Group B. V25 for left heart was 8.91% in Group A and 9.25% in Group B (p=0.77). V20 for ipsilateral lung was 21.67% in Group A and 25.65% in Group B (p=0.61) (Table 1). A total of 21 (70%) patients in Group A and 23 (76.6%) patients in Group B (P=0.12) were found to be Grade 1 according to the Eastern Cooperative Oncology Group (ECOG) Performance Status Scale (Table 1). 

**Table 1:** Patient characteristics and treatment protocol BMI: body mass index; ECOG: Eastern Cooperative Oncology Group; NACT: neoadjuvant chemotherapy; V25: volume receiving 25 Gy; V20: volume receiving 20 Gy; I/L: Ipsilateral *Data presented as Mean ± SD or Number of patients (Percentage)

Variables	Group A (30 patients)*	Group B (30 patients)*	P value
Age (years)	53.28 ± 9.73	55.67 ± 10.41	0.82
BMI (kg/m^2^)	21.56 ± 1.23	22.3 ± 1.65	0.79
NACT	17 (56.6%)	15 (50%)	0.56
Adjuvant chemotherapy	27 (90%)	28 (93.3%)	0.91
Hormonal therapy	19 (63.3%)	18 (60%)	0.87
V25 left heart (%)	8.91	9.25	0.77
V20 I/L lung (%)	21.67	25.65	0.61
ECOG Performance Status Scale score			
0	5 (16.6%)	3 (10%)	0.12
1	21 (70%)	23 (76.6%)	
2	4 (13.4%)	4 (13.4%)	

Table 2 depicts that 19 (63.4%) patients in Group A and 16 (53.4%) patients in Group B had a KPS score of 90 (p=0.73). The right breast was involved in 13 (43.4%) patients in Group A and 14 (46.6%) in Group B, and the left breast in 17 (56.6%) in Group A and 16 (53.4%) in Group B (p=0.81). Most of the patients were menopausal; 24 (80%) in Group A and 25 (83.4%) in Group B (p=0.91) (Table 2). Thirteen (43.4%) patients in Group A were ER-positive and PR-positive while this was true for 11 (36.66%) in Group B. Seven (23.4%) patients in Group A and four (13.4%) patients in Group B were triple positive (p=0.12) (Table 2). Eleven (36.6%) patients were of stage T2N1M0 in both groups. However, no statistical difference was observed in the TNM staging of both groups (p=0.26) (Table 2).

**Table 2:** Comparison of performance scores in both groups KPS: Karnofsky Performance Scale; ER: estrogen receptor; PR: progesterone receptor; HER 2: human epidermal growth factor receptor 2; TNM: tumor, nodes, metastases, (+): positive, (-): negative *Data presented as Number of patients (Percentage)

Parameters	Variables	Group A*	Group B*	P-value
KPS	70	2 (6.6%)	2 (6.6%)	0.73
	80	9 (30%)	12 (40%)	
	90	19 (63.4%)	16 (53.4%)	
Side	Right Breast	13 (43.4%)	14 (46.6%)	0.81
	Left Breast	17 (56.6%)	16 (53.4%)	
Menstrual status	Pre	6 (20%)	5 (16.6%)	0.91
	Post	24 (80%)	25 (83.4%)	
Modality	ER (+)/PR (-)/HER2 (-)	1 (3.4%)	3 (10%)	0.12
	ER (+)/PR (+)/ HER2 (-)	13 (43.4%)	11 (36.6%)	
	ER (+)/PR (-) /HER2 (+)	2 (6.6%)	6 (20%)	
	ER (-)/PR (+)/HER2 (+)	1 (3.4%)	5 (16.6%)	
	Triple (+)	7 (23.4%)	4 (13.4%)	
	Triple (-)	6 (20%)	1 (3.4%)	
TNM staging	T1N1M0	7 (23.4%)	1 (3.4%)	0.26
	T2N1M0	11 (36.6%)	11 (36.6%)	
	T2N2M0	3 (10%)	6 (20%)	
	T3N0M0	2 (6.6%)	7 (23.4%)	
	T3N1M0	7 (23.4%)	5 (16.6%)	

There was no significant difference between the two fractionation protocols with regard to baseline ejection fraction (EF). No ECG changes were observed in any patient of either group before or after the treatment. The mean pre-treatment and post-treatment EF in both Group A and Group B was 58%. The overall median decrease in EF didn’t exceed 5% in both groups (p=0.42) (Table 3).

**Table 3:** Ejection fraction (EF) of patients under Group A *Data presented as Number of patients (Percentage); p-value calculated by Chi-square test

EF (%)	Before treatment*	After treatment*	At 3 months*	At 6 months*	At 12 months*	P-value
55	3 (10%)	3 (10%)	3 (10%)	2 (6.6%)	3 (10%)	0.42
56	1 (3.4%)	2 (6.6%)	1 (3.4%)	2 (6.6%)	-	
57	2 (6.6%)	4 (13.4%)	4 (13.4%)	6 (20%)	7 (23.4%)	
58	6 (20%)	4 (13.4%)	6 (20%)	8 (26.6%)	8 (26.6%)	
59	8 (26.6%)	9 (30%)	8 (26.6%)	6 (20%)	7 (23.4%)	
60	8 (26.6%)	7 (23.4%)	7 (23.4%)	6 (20%)	5 (16.6%)	
61	1 (3.4%)	1 (3.4%)	1 (3.4%)	-	-	
62	1 (3.4%)	-	-	-	-	

There was no significant difference between the two fractionation protocols in regard to baseline Ejection Fraction (EF) (P= 0.08). Similar to Group A patients, no ECG changes were observed among the patients of Group B (Table 4).

**Table 4:** Ejection fraction (EF) of patients under Group B *Data presented as Number of patients (Percentage); p-value calculated by Chi-square test

EF (%)	Before treatment*	After treatment*	At 3 months*	At 6 months*	At 12 months*	P-value
55%	2 (6.6%)	2 (6.6%)	3 (10%)	4 (13.4%)	4 (13.4%)	0.08
56%	3 (10%)	2 (6.6%)	1 (3.4%)	1 (3.4%)	1 (3.4%)	
57%	2 (6.6%)	5 (16.6%)	5 (16.6%)	5 (16.6%)	7 (23.4%)	
58%	4 (13.4%)	5 (16.6%)	6 (20%)	6 (20%)	7 (23.4%)	
59%	8 (26.6%)	8 (26.6%)	6 (20%)	7 (23.4%)	9 (30%)	
60%	7 (23.4%)	5 (16.6%)	8 (26.6%)	5 (16.6%)	1 (3.4%)	
61%	2 (6.6%)	2 (6.6%)	-	1 (3.4%)	-	
62%	2 (6.6%)	1 (3.4%)	1 (3.4%)	1 (3.4%)	1 (3.4%)	

On comparing the variation in EF at different intervals of toxicity assessment in Group A, no significant difference was observed (Table 5). Similarly, no significant difference was observed while comparing the variation in EF at different intervals of toxicity assessment in Group B (Table 6).

**Table 5:** Variation in ejection fraction at different intervals of toxicity assessment in Group A *Data presented as Number of patients (Percentage); p-value calculated by Chi-square test

Change	Completion of treatment*	At 3 months*	At 6 months*	At 12 months*	P-value
No change	28 (93.4%)	16 (53.4%)	16 (53.4%)	16 (53.4%)	0.06
-1%	1 (3.4%)	7 (23.4%)	9 (30%)	9 (30%)	
-2%	1 (3.4%)	7 (23.4%)	5 (16.6%)	4 (13.4%)	
-3%	0 (0%)	0 (0%)	0 (0%)	1 (3.4%)	
-4%	0 (0%)	0 (0%)	0 (0%)	0 (0%)	
-5%	0 (0%)	0 (0%)	0 (0%)	0 (0%)	

**Table 6:** Variation in ejection fraction at different intervals of toxicity assessment in Group B *Data presented as Number of patients (Percentage); p-value calculated by Chi-square test

Change	Completion of treatment*	At 3 months*	At 6 months*	At 12 months*	P-value
No change	18 (60%)	18 (60%)	13 (43.4%)	10 (33.4%)	0.25
+1%	1 (3.4%)	2 (6.6%)	3 (10%)	2 (6.6%)	
+2%	2 (6.6%)	1 (3.4%)	0 (0%)	0 (0%)	
-1%	6 (20%)	5 (16.6%)	8 (26.6%)	14 (46.7%)	
-2%	1 (3.4%)	2 (6.6%)	6 (20%)	2 (6.6%)	
-3%	1 (3.4%)	1 (3.4%)	0 (0%)	2 (6.6%)	
-4%	1 (3.4%)	1 (3.4%)	0 (0%)	0 (0%)	
-5%	0 (0%)	0 (0%)	0 (0%)	0 (0%)	

In Group A, the pre-treatment mean value of FEV1 (in liters) was 2.57±0.21 and the post-treatment value of FEV1 was 2.53±0.19, and at three, six, and 12 months follow-up, it was 2.47±0.18, 2.38±0.18, and 2.17±0.18, respectively (p=0.44) (Table 7). The pre-treatment FVC was 3.08±0.24, which decreased to 2.78±0.25 by the 12-month follow-up (p=0.07). Similarly, no significant results were obtained while comparing FEV1/FVC from pre-treatment up to the 12-month follow-up (p=0.09) (Table 7). 

**Table 7:** Pulmonary function tests in Group A FEV_1_: forced expiratory volume in one second; FVC: forced vital capacity *Data presented as Mean ± Standard Deviation; p-value calculated by ANOVA

Pulmonary function test	Pre-treatment*	Post-treatment*	Follow-up at 3 months*	Follow-up at 6 months*	Follow-up at 12 months*	P-value
FEV_1_(L)	2.57±0.21	2.53±0.19	2.47±0.18	2.38±0.18	2.17±0.18	0.44
FVC (L)	3.08±0.24	2.98±0.25	2.96±0.21	2.84±0.422	2.78±0.25	0.07
FEV_1_/FVC	83.68±2.80	84.84±2.55	83.43±1.77	83.71±2.94	82.99±6.42	0.09

In Group B, the pre-treatment mean value of FEV1 (liters) was 2.68±0.40 and the post-treatment mean value of FEV1 was 2.53±0.38, which gradually decreased over a period of 12 months to 2.25±0.39 (p=0.14). The mean FVC at pre-treatment was 3.18±0.50, post-treatment was 3±0.49, and at follow-ups at three, six, and 12 months, it was 2.98±0.51, 3.0±0.42, and 2.84±0.42, respectively (p=0.16) (Table 8). No statistical significance was observed in the ratio of FEV1/FVC on comparing the pre-treatment mean value with post-treatment and follow-up values (p=0.97) (Table 8).

**Table 8:** Pulmonary function test in Group B FEV_1_: forced expiratory volume in one second; FVC: forced vital capacity *Data presented as Mean ± Standard Deviation; p-value calculated by ANOVA

Pulmonary Function test	Pre-treatment*	Post-treatment*	Follow-up at 3 months*	Follow-up at 6 months*	Follow-up at 12 months*	P-value
FEV_1 _(L)	2.68±0.40	2.53±0.38	2.43±0.37	2.39±0.38	2.25±0.39	0.14
FVC (L)	3.18±0.50	3.0±0.49	2.98±0.51	3.0±0.42	2.84±0.42	0.16
FEV_1_/FVC	84.41±4.85	84.37±4.42	81.99±5.12	79.52±4.32	79.18±6.35	0.97

Response evaluation in Group A reveals that 22 patients had complete responses a week after completion of treatment while six patients had a partial response, and two patients had stable disease. At the three-month follow-ups, no change in patients' responses was observed. At six months, 21 patients had a complete response, one patient had progressive disease, six patients had a partial response, and only two patients had stable disease. A similar response was observed after 12 months of follow-up (p=0.53) (Table 9).

**Table 9:** Response to treatment in Group A *Data presented as Number of patients (Percentage);  p- value calculated by Chi-square test

Response	After treatment*	At 3 months*	At 6 months*	At 12 months*	P-value
Complete response	22 (73.4%)	22 (73.4%)	21 (70.0%)	21 (70.0%)	0.53
Progressive disease	-	-	1 (3.4%)	1 (3.4%)	
Partial response	6 (20%)	6 (20%)	6 (20%)	6 (20%)	
Stable disease	2 (6.6%)	2 (6.6%)	2 (6.6%)	2 (6.6%)	
Total	30 (100%)	30 (100%)	30 (100%)	30 (100%)	

In Group B, after treatment 22 patients had a complete response, one patient had progressive disease, six patients had a partial response, and one patient had stable disease. At the three-month follow-up, 22 patients showed complete response, five patients showed partial response, and three patients showed stable disease. On further follow-ups at six and 12 months, a similar response was found as at the three-month follow-up (p=0.64) (Table 10).

**Table 10:** Response to treatment in Group B *Data presented as Number of patients (Percentage); p-value calculated by Chi-square test

Response	After treatment*	At 3 months*	At 6 months*	At 12 months*	P-value
Complete response	22 (73.4%)	22 (73.4%)	22 (73.4%)	22 (73.4%)	0.64
Progressive disease	1 (3.4%)	-	-	-	
Partial response	6 (20%)	5 (16.6%)	5 (16.6%)	5 (16.6%)	
Stable disease	1 (3.4%)	3 (10%)	3 (10%)	3 (10%)	
Total	30 (100%)	30 (100%)	30 (100%)	30 (100%)	

## Discussion

This study analyses whether a widely accepted shortened (altered fractionation) regimen used to treat women with breast cancer can be as effective as conventionally used longer fractionation regimes. In a study conducted by Yarnold et al., two altered fractionation regimens of 39 Gy in 13 fractions and 42.9 Gy in 13 were used [8]. In Whelan's 2002 study, a hypo-fractionation regimen of 42.5 Gy in 16 fractions was compared with a conventional regimen of 50 Gy in 25 fractions [9]. In the hypo-fractionation schedule of Whelan et al.'s study, biological effective dose (BED) was 53.76 Gy (α/β=10, D=42.5 Gy, d=2.65 Gy, and n=16) while in conventional fractionation, it was 60 Gy (α/β=10, D=50 Gy, d=2 Gy and n=25) [9], where D = total dose; d = dose per fraction; and the α/β value will be related to the tissue on which it is desired to estimate the effect. In the present study, if α/β = 10, the BED is 50 Gy (α/β=10, D=40 Gy, d=2.5 Gy, and n=16).

It should be emphasized that long‑term follow‑up of the Standardization of Breast Radiotherapy (START) trials confirmed that the appropriately dosed hypo-fractionated radiotherapy is safe and effective for patients with early-detected breast cancer. In particular, the results of the START trials revealed that follow‑up was still short for cardiovascular events [9]. Moreover, some studies suggest that hypo-fractionated breast radiotherapy might be safer for the heart than conventional regimens [10]. In the present study, we have also compared the toxicity profile of patients receiving conventional radiation doses and hypo-fractionated doses of radiotherapy, which suggests no significant difference between both fractionation schedules in terms of cardiac toxicity.

Our results showed that although follow-up was short for cardiac events, there was no major difference between the toxicity profiles in studies with long-term follow-up. Conventionally, cardiotoxicity is monitored by measuring the LV EF [11]. It may include an EF decline of 20%, a decrease of LV EF by 10 points to 55%, or a drop of LV EF by 45% [12]. Nearly all patients with systolic dysfunction have more or less a degree of concomitant diastolic dysfunction, especially impaired relaxation, and variable decreases in ventricular compliance. In the study of Jacob et al., the prevalence of diastolic dysfunction in asymptomatic patients after mediastinal radiation was 14% [13]. A multidisciplinary novel approach to early detection of radiation-induced cardiotoxicity, the breast cancer and cardiotoxicity induced by radiotherapy (BACCARAT) project, was based on an early-stage clinical study, which suggests that the long-term significance of the observed changes in echocardiography is an important issue and can be used as an efficient tool for the prediction of cardiac toxicity [13]. In our study, we have also used findings of 2D echocardiography as a tool for the assessment of cardiac toxicity.

In the long-term results of a study done by Whelan et al., worsening of the cosmetic outcome over time was observed, which coincided with the increase in toxic effects of irradiation of the skin and subcutaneous tissue [9]. Although older age and large tumor size were associated with a worse cosmetic outcome, the outcomes of the hypo-fractionated regimen were similar to those of the standard regimen. So, a longer study with a larger sample size may have provided a better overview of both early and late toxicity events with survival outcomes, which may guide us towards better treatment regimens both in terms of toxicity and outcome. Another limitation of our study was that tumor analysis in terms of disease-free survival and overall survival has not been estimated in the study, which may provide better credibility to results. 

## Conclusions

Hypo-fractionated radiotherapy or conventional radiotherapy can be used as an alternative method following breast cancer surgery. Moreover, the outcome of both regimens revealed no significant differences in terms of pulmonary toxicity, cardiac toxicity, and response. This indicates that a shorter duration of hypo-fractionated radiotherapy can also be implemented in breast cancer patients. Hypo-fractionated radiotherapy may improve the patient turnover rate, timely follow-up, and decrease the overall cost of treatment.
